# Atypical Presentation of Massive Pulmonary Embolism Leading to In-Hospital Cardiac Arrest and Successful Thrombolysis

**DOI:** 10.7759/cureus.113380

**Published:** 2026-07-25

**Authors:** Humaid Sadiq, Falaah Abdul Hameed, Antria Jenifer, Sonia Lamichhane, Mansoor M Husain

**Affiliations:** 1 Emergency Medicine, Tawam Hospital, Al Ain, ARE

**Keywords:** atypical presentation, cardiac arrest, in-hospital cardiac arrest, massive pulmonary embolism, obstructive shock, thrombolysis

## Abstract

Pulmonary embolism (PE) is a potentially fatal condition with a broad spectrum of clinical presentations, many of which are non-specific and may delay diagnosis, particularly in patients without classical risk factors. Although dyspnea, chest pain, and tachycardia are considered typical features, atypical symptoms such as dizziness, palpitations, and syncope can obscure early recognition and contribute to adverse outcomes. We describe a woman in her early 40s with a background of anemia and colonic disease who presented with dizziness, palpitations, and lethargy. Initial assessment revealed persistent tachycardia, tachypnea, metabolic acidosis, and elevated troponin and D-dimer levels. Despite early fluid resuscitation, the patient deteriorated rapidly with hemodynamic instability. Computed tomography pulmonary angiography confirmed a massive PE. She subsequently suffered a cardiac arrest and achieved return of spontaneous circulation following systemic thrombolysis administered during cardiopulmonary resuscitation. Her clinical course was complicated by catastrophic intra-abdominal hemorrhage, likely secondary to thrombolytic therapy, necessitating emergency laparotomy and intensive care unit admission. Planned catheter-directed thrombectomy was abandoned because of active bleeding and hemodynamic instability. She developed multiorgan failure requiring vasopressor support and continuous renal replacement therapy. Further complications included radial artery thrombosis requiring surgical thrombectomy, recurrent pulmonary edema, and non-ST-segment elevation myocardial infarction. The occurrence of recurrent arterial and venous thrombotic events raised suspicion for antiphospholipid syndrome as an underlying prothrombotic condition. Following a prolonged and complex hospital course involving multiple intensive care unit readmissions and surgical interventions, the patient recovered and was discharged after 33 days on anticoagulation with planned multidisciplinary follow-up. This case highlights the importance of maintaining a high index of suspicion for PE in patients presenting with atypical symptoms and underscores the need to consider underlying thrombophilia in severe or recurrent thrombotic disease.

## Introduction

Pulmonary embolism (PE) is a common and potentially life-threatening manifestation of venous thromboembolism, accounting for significant morbidity and mortality worldwide. Early diagnosis remains challenging because clinical presentation is highly variable and often non-specific. Although dyspnea, pleuritic chest pain, tachycardia, and hypoxemia are considered classic features, many patients present with atypical symptoms that may delay recognition and treatment [1-3]. Diagnostic algorithms incorporating clinical probability assessment and D-dimer testing have improved the evaluation of suspected PE; however, diagnostic uncertainty may persist, particularly in patients without obvious risk factors or with atypical presentations [1,2].

Massive, or high-risk, PE is characterized by hemodynamic instability and carries a particularly poor prognosis if not identified and treated promptly [1,3]. The pathophysiology involves acute obstruction of the pulmonary vasculature, resulting in right ventricular strain, reduced cardiac output, and potentially rapid progression to obstructive shock and cardiac arrest [1,3]. Because compensatory mechanisms may initially preserve oxygenation and mask the severity of disease, patients can present with non-specific symptoms before sudden clinical deterioration occurs [3].

Underlying thrombophilia disorders should also be considered in patients presenting with severe, unexplained, or recurrent thromboembolic events. Antiphospholipid syndrome (APS) is a well-recognized acquired thrombophilia associated with both venous and arterial thrombosis and may manifest with recurrent or catastrophic thromboembolic disease [4,5]. Recognition of APS has important implications for long-term management, anticoagulation strategy, and the prevention of recurrent events [4,5].

We report a case of massive PE presenting atypically with dizziness, palpitations, and lethargy in a woman with a background of anemia and colonic disease. The case was further complicated by cardiac arrest requiring thrombolysis, catastrophic intra-abdominal hemorrhage, multiorgan failure, and recurrent thrombotic complications, highlighting the diagnostic challenges and therapeutic complexities associated with high-risk PE.

## Case presentation

Initial presentation

A middle-aged woman in her early 40s with known comorbidities of anemia and a possible history of colonic disease presented to the emergency department with a chief complaint of dizziness. History was primarily obtained from her husband, as the patient appeared lethargic at presentation. She had been experiencing dizziness for approximately seven hours prior to arrival, associated with chest discomfort, three episodes of vomiting, and generalized weakness. She also reported intermittent palpitations over the preceding three months. Review of systems was otherwise negative, including no gastrointestinal symptoms such as hematemesis, melena, hematochezia, abdominal pain, or diarrhea; no respiratory symptoms such as shortness of breath; no neurological symptoms such as loss of consciousness or visual disturbances; no genitourinary symptoms such as dysuria; and no infectious or systemic symptoms such as fever. However, she reported feeling anxious in recent weeks in relation to an upcoming Quran memorization competition. There were no recent travel history or sick contacts and no prior similar episodes except for palpitations. Her obstetric history was Gravida 4, Para 4, Abortions 0.

Emergency department assessment

On arrival, her vital signs were as follows: blood pressure 100/80 mmHg, heart rate 126 beats per minute, respiratory rate 30 breaths per minute, temperature 36.8°C, and oxygen saturation 99% on room air. Given her borderline hemodynamic stability, she was immediately transferred from the triage area to the resuscitation bay, where all subsequent assessment and management were performed. On examination, she appeared pale and acutely unwell, with a Glasgow Coma Scale (GCS) score of 15/15. Chest examination revealed equal bilateral air entry with no wheeze or crepitations. Cardiovascular examination demonstrated weak peripheral pulses with normal S1 and S2 heart sounds, without murmurs or peripheral edema. Abdominal examination revealed a soft abdomen with right lower quadrant tenderness but no guarding or rebound tenderness. Neurological examination revealed no focal deficits, with grossly intact motor and sensory function.

Initial investigations

**Figure 1:**
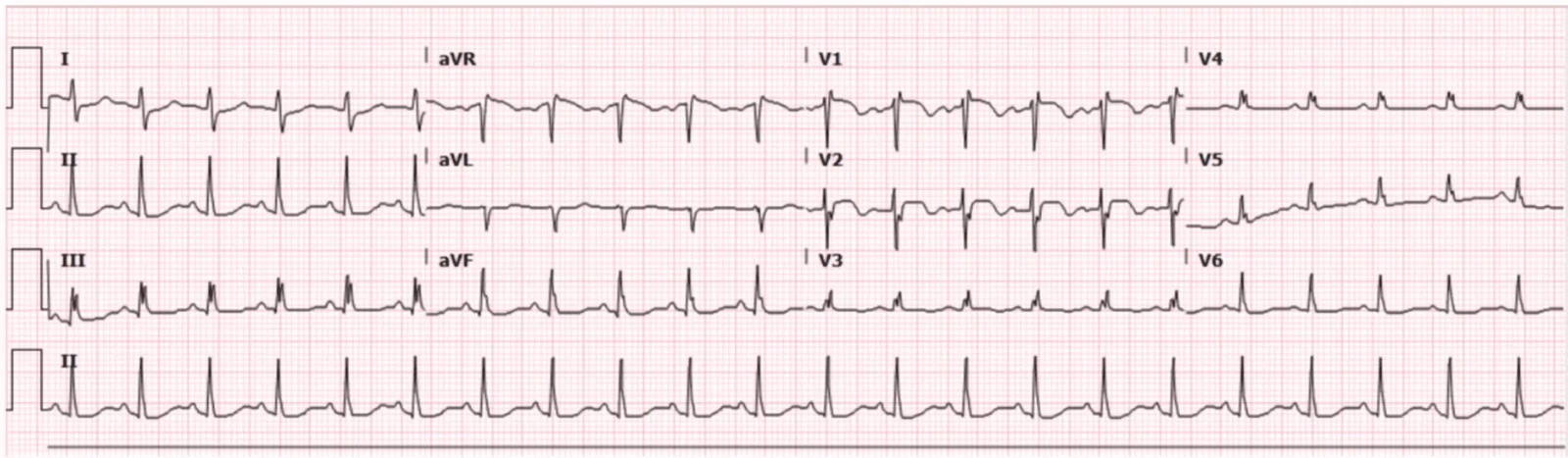
Electrocardiogram (ECG) demonstrating sinus tachycardia

Electrocardiogram (ECG) demonstrated sinus tachycardia (Figure 1), and chest X-ray was unremarkable.

Initial venous blood gas revealed non-anion gap metabolic acidosis with hyperlactatemia (lactate 4.8 mmol/L) and a normal hemoglobin level of 136 g/L, with pH 7.30, pCO₂ 35.1 mmHg, HCO₃ 17 mmol/L, base excess -8.5, anion gap 13, and glucose 10.5 mmol/L. Her creatinine was mildly elevated at 97.3 mmol/L (reference range: 44.2-88.4 mmol/L), and C-reactive protein (CRP) was elevated at 26.7 mg/L (≤5.0 mg/L) (Table 1).

**Table 1:** Initial blood investigation results

Test	Result	Reference range	Unit
pH	7.30	7.35-7.45	-
pCO₂	35.1	35.0-45.0	mmHg
pO₂	41.0	25.0-40.0	mmHg
HCO₃	17	22-26	mmol/L
Base excess (BE)	-8.5	-2.0-2.0	mmol/L
Total hemoglobin (THB)	136	120-170	g/L
Carboxyhemoglobin (COHB)	1.2	0.0-2.0	%
Potassium (K)	4.2	3.4-5.1	mmol/L
Chloride (Cl)	111	98-107	mmol/L
Sodium (Na)	141	136-145	mmol/L
Glucose (Glu)	10.5	3.9-6.0	mmol/L
Lactate (Lac)	4.8	0.5-2.2	mmol/L
Creatinine	97.3	44.2-88.4	mmol/L
C-reactive protein (CRP)	26.7	≤5.0	mg/L

The remainder of her laboratory workup, including complete blood count, liver function tests, and basic metabolic panel, was within normal limits. A full blood workup including cardiac biomarkers and D-dimer was sent.

Initial management and clinical deterioration

Following the initial assessment at time 0, two intravenous lines were established, and the patient received 1 L of intravenous normal saline along with 1 g intravenous paracetamol. Despite these measures, she remained tachycardic (124-126 beats per minute) and tachypneic (respiratory rate 34 breaths per minute) while maintaining oxygen saturation at 97% on room air. A second 1 L bolus of intravenous normal saline was administered.

Approximately 30 minutes after presentation, her clinical condition deteriorated with hypotension measuring 80/60 mmHg and new-onset central chest pain. Repeat assessment revealed a significant inter-arm blood pressure difference, with the right arm measuring 80/60 mmHg and the left arm 100/70 mmHg. An additional 500 mL intravenous normal saline bolus was administered using a pressure bag. Further investigations, including serum β-hCG, serum ketones, and crossmatch for two units of packed red blood cells, were sent. A right radial arterial line was inserted, confirming persistent hypotension at 84/62 mmHg, and norepinephrine infusion was commenced at 5 µg/min.

Diagnostic escalation

Approximately two hours after presentation, laboratory results became available and demonstrated markedly elevated troponin-I (1489.9 ng/L) and D-dimer (>35.2 mg/L), significantly raising suspicion for a massive PE. Repeat arterial blood gas analysis showed worsening metabolic acidosis with pH 7.14, pCO₂ 24.4 mmHg, HCO₃ 8 mmol/L, base excess -19.0, anion gap 12, glucose 14.8 mmol/L, and lactate 7.6 mmol/L (Table 2). Serum β-ketones were negative, making diabetic ketoacidosis unlikely.

**Table 2:** Arterial blood gas and subsequent lab results

Test	Result	Reference range	Unit
pH	7.14	7.35-7.45	-
pCO₂	24.4	35.0-45.0	mmHg
pO₂	200.0	25.0-40.0	mmHg
HCO₃	8	22-26	mmol/L
Base excess (BE)	-19.0	-2.0-2.0	mmol/L
Total hemoglobin (THB)	121	120-170	g/L
Carboxyhemoglobin (COHB)	0.7	0.0-2.0	%
Potassium (K)	4.9	3.4-5.1	mmol/L
Chloride (Cl)	119	98-107	mmol/L
Sodium (Na)	139	136-145	mmol/L
Glucose (Glu)	14.8	3.9-6.0	mmol/L
Lactate (Lac)	7.6	0.5-2.2	mmol/L
D-dimer	>35.2	0.219-0.523	mg/L
Troponin-I	1489.9	≤15.60	ng/L

**Figure 2:**
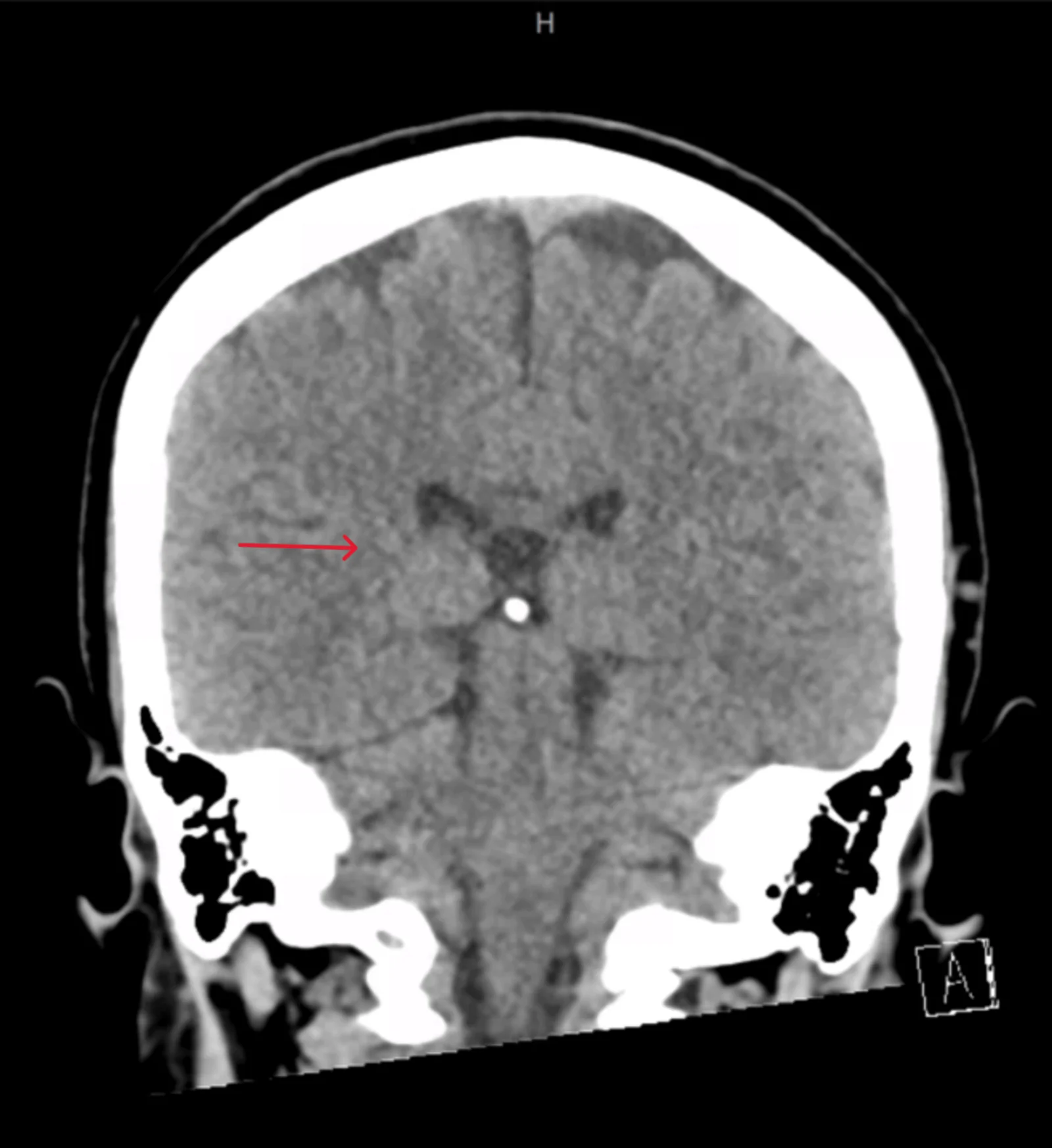
Non-contrast computed tomography (CT) of the head demonstrating no acute intracranial pathology The straight arrow indicates a normal anatomical landmark for orientation.

**Figure 3:**
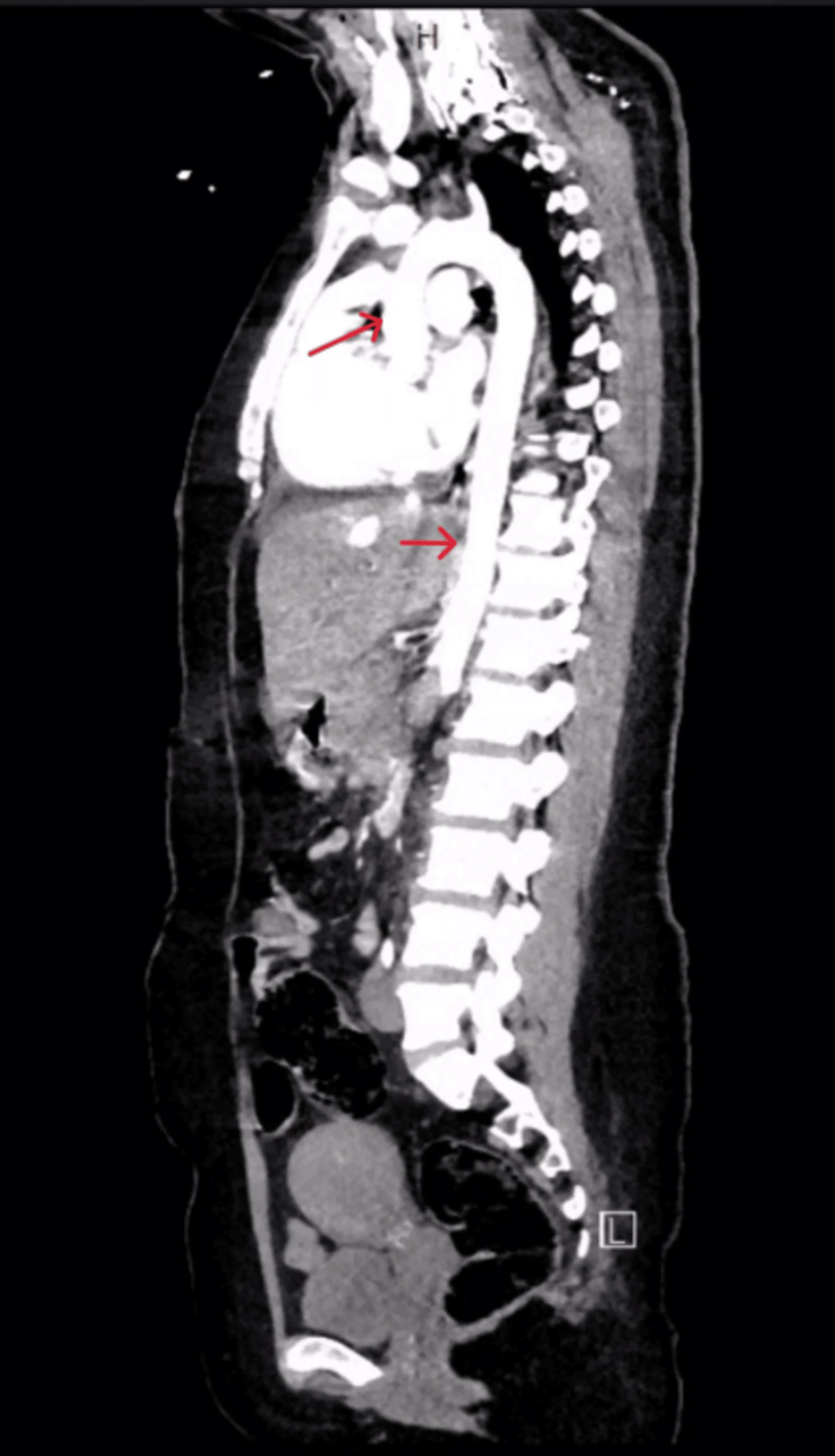
Computed tomography angiography (CTA) of the chest and abdomen demonstrating a patent and well-opacified thoracic aorta with contrast, with no aortic dissection, aneurysmal dilatation, significant stenosis, or occlusion (red arrows)

**Figure 4:**
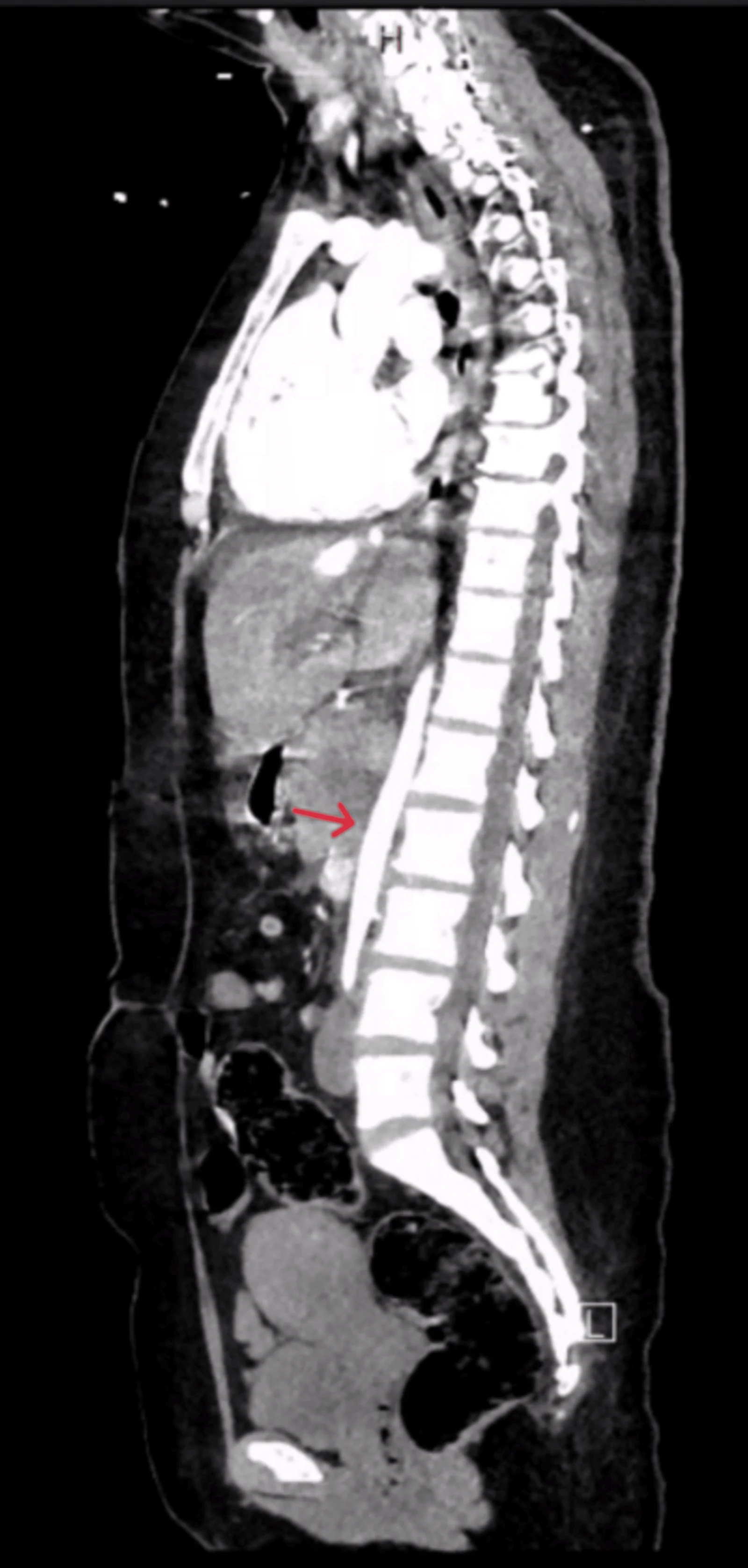
Computed tomography angiography (CTA) of the chest and abdomen demonstrating a patent and well-opacified abdominal aorta with contrast, with no aortic dissection, aneurysmal dilatation, significant stenosis, or occlusion (red arrow)

Given the rapidly worsening clinical picture, the combination of refractory hypotension, markedly elevated D-dimer and troponin levels, and the newly identified inter-arm blood pressure difference, both massive PE and acute aortic syndrome were considered important differential diagnoses. The patient was urgently transferred for non-contrast computed tomography (CT) of the head, CT pulmonary angiography (CTPA), and CT angiography (CTA) of the chest and abdomen. All imaging studies were completed during the same CT session. The CT of the head demonstrated no acute intracranial abnormality (Figure 2), while CTA of the chest and abdomen showed no evidence of acute aortic syndrome (Figures 3-4). At that time, the formal CTPA interpretation was still pending. The intensive care unit (ICU) team was simultaneously informed. Given the undifferentiated presentation and ongoing evaluation for alternative life-threatening causes of chest pain and shock, including acute coronary syndrome and aortic dissection, anticoagulation was deferred pending definitive imaging.

Cardiac arrest and thrombolysis

**Figure 5:**
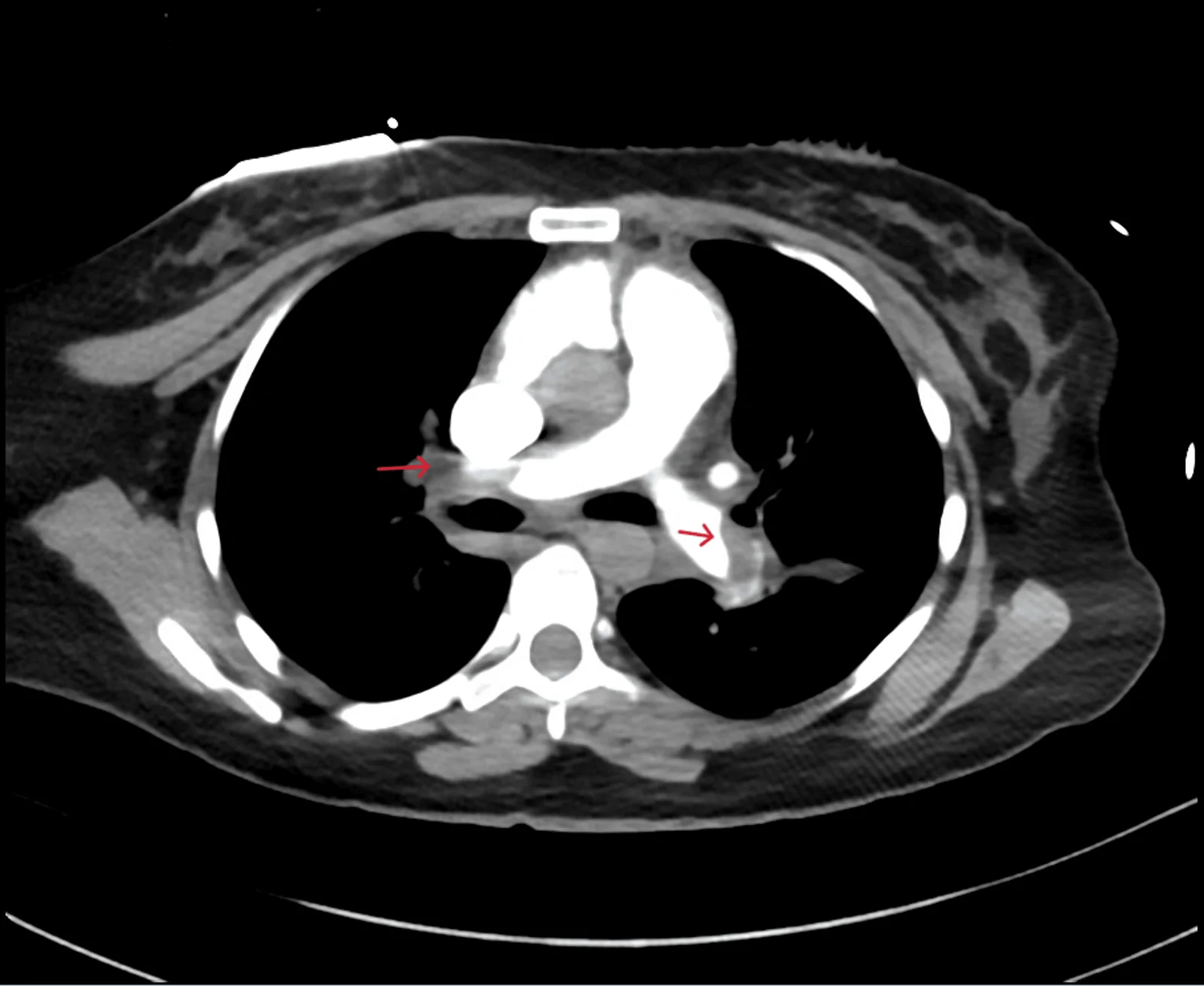
Computed tomography pulmonary angiography (CTPA) demonstrating bilateral filling defects within the right and left main pulmonary arteries (red arrows)

**Figure 6:**
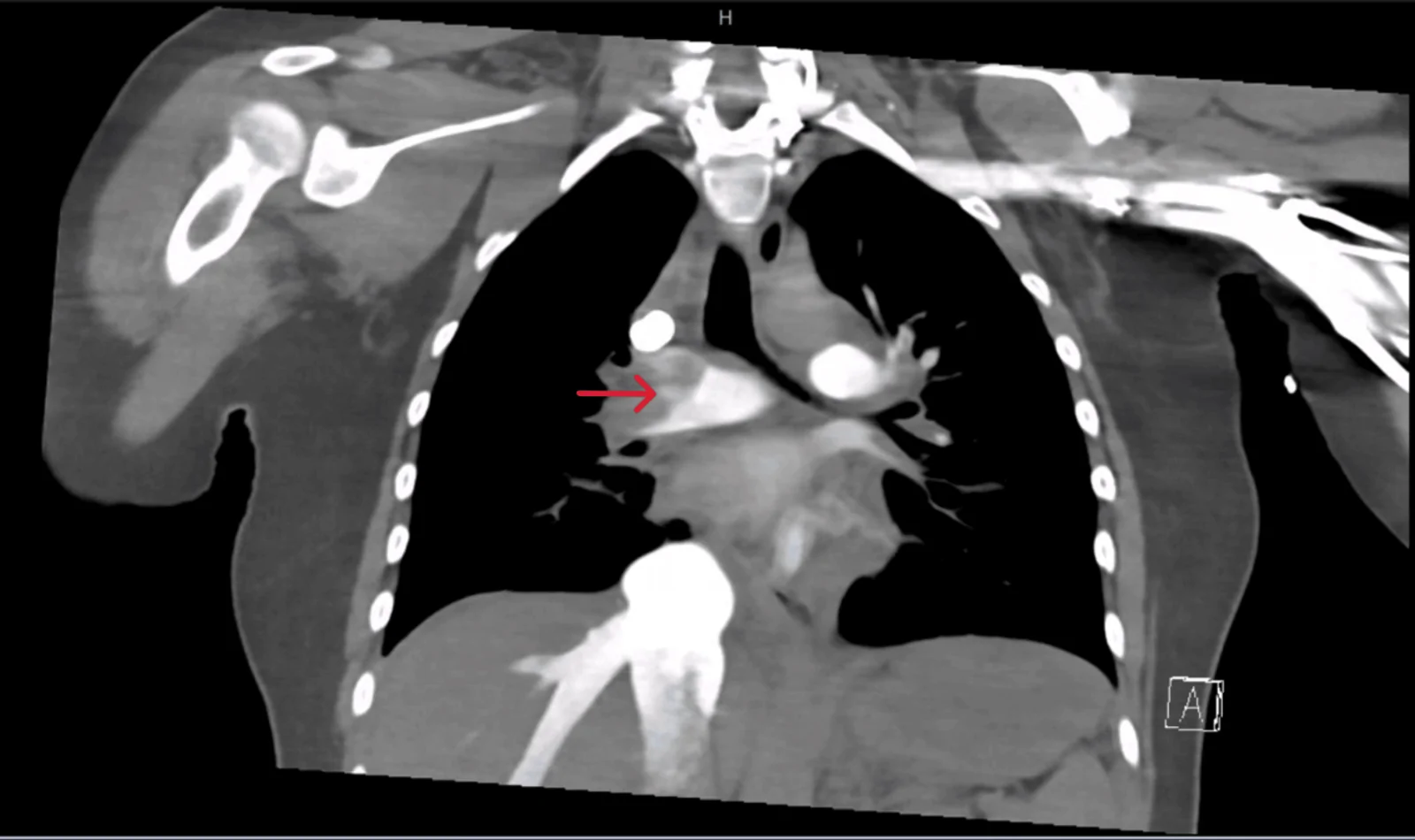
Computed tomography pulmonary angiography (CTPA) demonstrating extension of the embolus into the right main pulmonary artery (red arrow)

**Figure 7:**
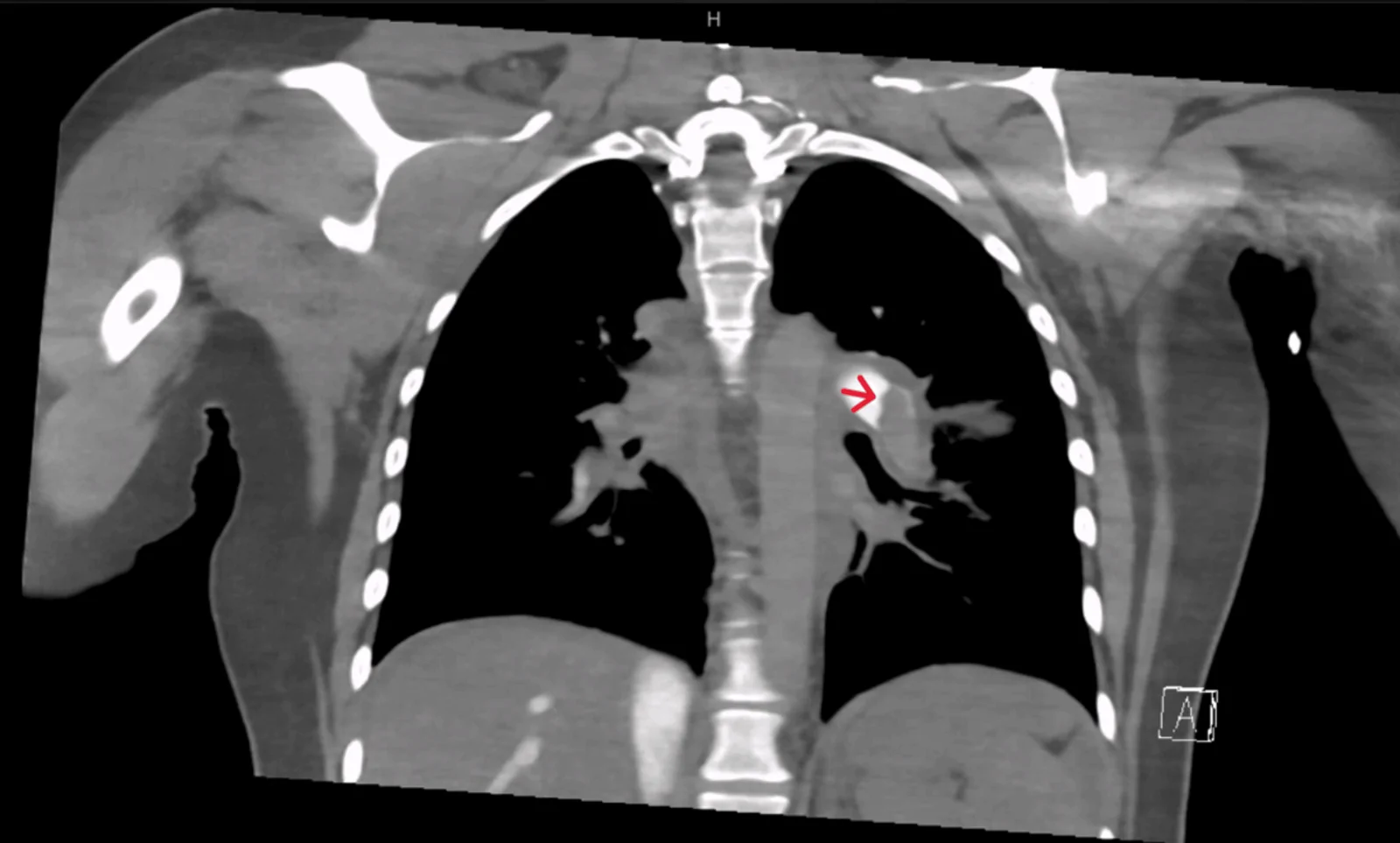
Computed tomography pulmonary angiography (CTPA) demonstrating extension of the embolus into the left main pulmonary artery (red arrow)

Approximately three hours after presentation, while being transferred back from the CT scanner to the resuscitation bay, the patient developed agonal respirations and rapidly progressed to cardiac arrest. Cardiopulmonary resuscitation (CPR) was initiated immediately in accordance with Advanced Cardiovascular Life Support (ACLS) guidelines. During ongoing resuscitation, while CPR was in progress, the radiologist provided an urgent verbal report of the previously performed CTPA confirming a massive saddle PE (Figures 5-7).

Intravenous alteplase was therefore immediately administered as a 50 mg bolus followed by 50 mg infusion over 20 minutes during CPR. Return of spontaneous circulation (ROSC) was achieved approximately three minutes later, after which post-resuscitation care was initiated.

Post-resuscitation management

Following ROSC, persistent hypotension required continuation of norepinephrine infusion; however, due to ongoing hemodynamic instability, vasopressor support was escalated to an epinephrine infusion, resulting in the stabilization of blood pressure at 125/86 mmHg. The patient underwent rapid sequence intubation using ketamine and rocuronium, followed by post-intubation sedation with fentanyl and midazolam. A right femoral central venous catheter was inserted for ongoing vasoactive support and hemodynamic monitoring.

Outcome and ICU course

Interventional radiology recommended mechanical thrombectomy once hemodynamically stable, and the ICU and anesthesia teams were involved in planning. During pulmonary angiography, bilateral filling defects consistent with PE were identified; however, clot burden appeared reduced compared to initial CTPA findings. During the procedure, the patient developed acute hypotension (76/45 mmHg) with a sudden drop in hemoglobin to 4.6 g/L. Focused assessment with sonography for trauma (FAST) revealed significant pelvic free fluid, prompting the activation of a massive transfusion protocol and initiation of vasopressor support. The procedure was aborted, and urgent CT imaging demonstrated active anterior upper abdominal venous bleeding.

The patient was transferred to the ICU and underwent emergency damage control surgery, during which approximately 2-3 L of intraperitoneal blood was evacuated. Bleeding from small sub-diaphragmatic veins anterior to the liver was identified and controlled with intra-abdominal packing, after which she returned to the ICU. Lower limb Doppler ultrasound showed no evidence of deep vein thrombosis. Due to worsening renal function and oliguria, continuous renal replacement therapy (CRRT) was initiated. A planned relook laparotomy was performed two days later for pack removal, hemostasis, and lavage.

ICU course and complications

During ICU stay, the patient developed right thumb ischemia secondary to a radial artery thrombus and underwent open thrombectomy. Multidisciplinary discussion identified a high thrombotic risk, and unfractionated heparin was initiated. She was extubated on day 8 after admission and transitioned off CRRT to intermittent renal support. Subsequently, she developed acute pulmonary edema requiring re-intubation and ICU readmission. Elevated troponin levels and new ECG changes were consistent with non-ST-segment elevation myocardial infarction (NSTEMI), and dual antiplatelet therapy was initiated. Echocardiography demonstrated global left ventricular systolic dysfunction with an ejection fraction of 20-25%. CT of the chest excluded recurrent PE but revealed bilateral pleural effusions and basal consolidation. Coronary angiography showed normal coronary arteries. CRRT was recommenced, vascular access was revised, and antibiotics were escalated to meropenem.

Following further recovery, renal function improved, and CRRT was discontinued. She was subsequently extubated but experienced a recurrent episode of pulmonary edema with chest pain requiring brief re-intubation and re-initiation of CRRT. She eventually stabilized with full recovery of respiratory function.

Outcome and discharge

The patient was eventually discharged after a 33-day hospital stay on therapeutic enoxaparin (Clexane) and bisoprolol, with outpatient follow-up arranged with cardiology, nephrology, and hematology services.

## Discussion

Massive PE is a critical medical emergency, defined by sustained hypotension (systolic blood pressure <90 mmHg), pulselessness, or persistent bradycardia, and is associated with high mortality if not promptly diagnosed and treated [1]. We present this case describing an atypical clinical presentation of massive PE leading to in-hospital cardiac arrest, with ROSC following thrombolysis. This was complicated by intra-abdominal bleeding, likely attributable to the combined effects of vigorous manual CPR and systemic thrombolytic therapy.

Retrospectively, the patient's clinical features would have yielded a moderate pre-test probability of PE based on the Wells score. However, the atypical early presentation with preserved oxygen saturation and non-specific symptoms broadened the initial differential diagnosis.

The patient's initial presentation was notably non-specific, including dizziness, vomiting, and chest discomfort, with respiratory distress at presentation (respiratory rate approximately 30/min), in the absence of initial hypoxemia. Such atypical presentations are increasingly recognized and remain a major contributor to diagnostic delay [1,2].

Persistent sinus tachycardia, elevated lactate, weak peripheral pulses, and subsequent hypotension suggested evolving obstructive shock despite initially preserved oxygen saturation. This pattern reflects early hemodynamic compromise in massive PE, where compensatory mechanisms may maintain oxygenation until abrupt circulatory collapse occurs [1,5].

Given the evolving clinical picture and the presence of markedly elevated D-dimer and troponin levels, urgent imaging was pursued to evaluate for life-threatening causes of shock, including PE and acute aortic syndrome. CTPA subsequently confirmed a high-risk (massive) PE [1,2]. The patient's hemodynamic status rapidly deteriorated with persistent hypotension requiring vasopressor support, culminating in cardiac arrest before definitive reperfusion therapy could be initiated.

An important consideration in this case is the potential contribution of APS as an underlying prothrombotic condition. APS is a well-recognized acquired thrombophilia associated with both venous and arterial thromboembolism, including recurrent and occasionally catastrophic PE. Current antithrombotic guidelines emphasize that patients with unexplained or disproportionate thrombotic events, particularly in the presence of anemia, inflammatory conditions, or gastrointestinal pathology, should prompt consideration of an underlying hypercoagulable state such as APS. Recognition of APS has important therapeutic implications, including the need for long-term anticoagulation and heightened vigilance for recurrent thromboembolic events. In high-risk patients, early suspicion and targeted evaluation for APS may facilitate earlier diagnosis and influence both acute and long-term management strategies [3,4].

For massive PE with hemodynamic instability or cardiac arrest, thrombolytic therapy is indicated and has been associated with improved rates of ROSC during CPR [1,6-9]. Evidence suggests that ROSC typically occurs within several minutes following administration during ongoing high-quality CPR [7,8]. The subsequent development of intra-abdominal hemorrhage highlights a well-recognized complication of CPR and thrombolysis [10,11]. CPR-associated liver injury occurs in a small but clinically significant proportion of cases.

Thrombolysis for PE-related cardiac arrest is not universally standardized, with decision-making influenced by bleeding risk and limited randomized evidence [12,13]. However, in life-threatening PE, contraindications are often considered relative given the extremely high mortality of untreated disease [13,14].

Following thrombolysis, the patient experienced complications consistent with systemic hypoperfusion and reperfusion injury after cardiac arrest [15]. Despite this, she survived with preserved neurological function and was discharged on anticoagulation with follow-up.

## Conclusions

This case highlights the challenges of diagnosing atypical massive PE, where non-specific symptoms delayed recognition and led to rapid cardiopulmonary collapse. Although thrombolysis during CPR was life-saving, it resulted in major hemorrhagic complications, underscoring the need for careful risk-benefit assessment. The patient's recovery despite multiorgan failure emphasizes the importance of early suspicion, multidisciplinary management, and close post-resuscitation monitoring in massive PE.
